# Noninvasive Imaging in Interventional Cardiology: Hypoplastic Left Heart Syndrome

**DOI:** 10.3389/fcvm.2021.637838

**Published:** 2021-02-01

**Authors:** Hannah Bellsham-Revell

**Affiliations:** Department of Paediatric Cardiology, Evelina London Children's Hospital, London, United Kingdom

**Keywords:** hypoplastic left heart syndrome, echocardiography, MRI, computed tomography, hybrid, Norwood, Glenn, Fontan

## Abstract

Hypoplastic left heart syndrome (HLHS) is a spectrum of left heart underdevelopment leaving the left side unable to support the systemic circulation. If active management is pursued, then the condition is managed with staged palliation to the Fontan circulation, leaving a systemic right ventricle. Through all surgical stages, and even after completion of Fontan, there are multiple areas that may require intervention, most frequently the branch pulmonary arteries which are essential to a successful Fontan circulation. Echocardiography is the mainstay of assessment, but there is an increasing use of magnetic resonance imaging (MRI) and computed tomography (CT) particularly in relation to extracardiac structures which can be more challenging with echocardiography. Both MRI and CT require set-up, experience and training, and usually sedation or anesthetic in smaller children, but can provide excellent imaging to guide interventions. Cardiac MRI is also able to quantify right ventricular (RV) function which can be challenging on echocardiography. This article describes the modalities available and their use in assessing patients with HLHS prior to catheter interventions.

## Introduction

**Figure 1:**
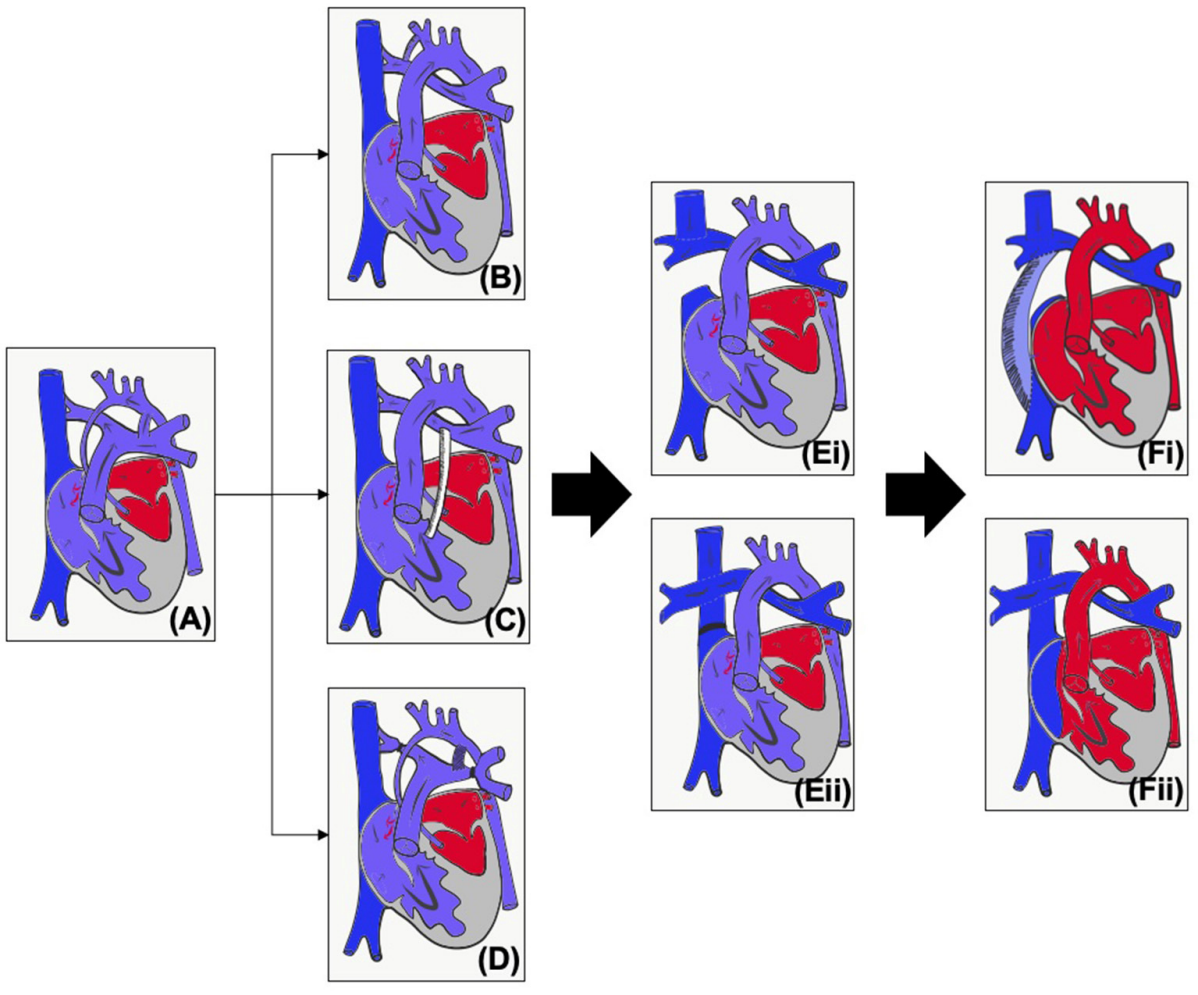
**(A)** Unoperated hypoplastic left heart syndrome. **(B)** Classical Norwood procedure. **(C)** Norwood procedure with Sano modification. **(D)** Hybrid procedure. **(E)** Superior cavopulmonary connection with (i) Glenn or (ii) hemi-Fontan. **(F)** Total cavopulmonary connection with (i) Fenestrated extracardiac conduit or (ii) Fenestrated lateral tunnel.

Occurring in around 1–2% of patients with congenital heart disease (1), hypoplastic left heart syndrome (HLHS) is universally fatal without intervention. Consisting of mitral stenosis/atresia and aortic stenosis/atresia with left ventricular (LV) hypoplasia (Figure 1A), the left heart is unable to support the systemic circulation. Although in some countries primary cardiac transplantation may be an option, due to low donor availability, the majority of patients, if the parents wish to proceed with active intervention, will undergo stage palliation through to the Fontan circulation (2).

The first operative procedure is performed in the first few days of life – the choice of initial palliation is based on institutional preference as well as risk factors such as prematurity and birth weight. The Norwood procedure (Figures 1B,C) consists of an atrial septectomy, reconstructing the aortic arch using the pulmonary artery, with anastomosis of the native aorta (Damus Kaye Stansel, DKS) and then providing pulmonary blood flow via either a modified Blalock-Taussig-Thomas shunt (BTTS) or a right ventricular to pulmonary artery (PA) conduit (Sano modification). The Norwood procedure (NP) carries a much more significant mortality compared to most other neonatal surgery, and this risk is increased for babies who are of low weight or premature (3, 4). The hybrid procedure (Figure 1D) was developed as a lower risk alternative which avoids cardiopulmonary bypass (5). Bands are placed on the branch pulmonary arteries and the baby is either maintained on a prostaglandin infusion or a stent inserted into the arterial duct. An intervention on the atrial septum may or may not be performed.

As caval flow increases and pulmonary vascular resistance decreases, by around 3–6 months of age infants are able to undergo a superior cavopulmonary connection (Figure 1E), leading to a more stable circulation. For those that have undergone the hybrid procedure, the next stage may be the NP (and then superior cavopulmonary connection, SCPC) or a comprehensive second stage, consisting of the arch reconstruction, DKS and atrial septectomy, but with a SCPC instead of an arterial or RV to PA shunt. Around 2–4 years of age, children will, if suitable, undergo the total cavopulmonary connection (TCPC, Figure 1F). Throughout staged palliation, catheter interventions may be required to optimize the circulation and prior to any intervention it is essential to properly assess the patient's anatomy and physiology, and to be able to plan the best intervention for the patient. Imaging can also be used to aid in medical decision making, but this paper concentrates on the use of imaging for intervention. The choice of imaging modality needs to be based on patient factors (e.g., compliance, acoustic windows) as well as the clinical question being asked (e.g., branch pulmonary arteries, tricuspid valve function) and balanced against risks of the modality (e.g., radiation, contrast). This article will review the modalities used in pre-intervention planning in HLHS as well as key considerations at each stage.

## Imaging Modalities

Noninvasive imaging modalities include echocardiography (two and three-dimensional), cardiac computed tomography (CT) and magnetic resonance imaging (MRI). Table 1 summarizes the main advantages and disadvantages of each. The clinical question must be carefully considered to ensure the correct modality is chosen, and in many cases multimodality imaging is undertaken. Echocardiography is the mainstay of assessment as it is readily available, and all cardiologists are proficient, however if CT and MRI are available then they should be considered in certain circumstances which will be described below.

**Table 1:** Strengths, weaknesses, and common uses of non-imaging for intervention in hypoplastic left heart syndrome.

	**2D and 3D Echo**	**CT**	**MRI**
Strengths	Readily available, anesthetic and sedation not usually required Good functional and physiological information on AV and VA valves Validated assessment tools for LV systolic and diastolic function Great vessel anatomy reasonably well seen in smaller children Can see flow in shunts and across atrial septum to interrogate for narrowing and look at secondary measures (e.g., pulmonary vein Dopplers in atrial restriction) No radiation	Very clear imaging of even very small vessels Allows assessment of airways and lung parenchyma Quick acquisition, so can be used in infants without sedation	“Gold standard” for function assessment Quantification of flow allowing calculation of collateral flow, valvar regurgitation etc. When combined with pressure data from catheter can be used to calculate pulmonary vascular resistance Can be done without the use of contrast
Weaknesses	Limited by acoustic windows Reliant on patient compliance Assessment of RV systolic and diastolic function challenging Great vessel imaging more challenging in older patients Arterial shunt flow can be seen, but difficult to define if subtle narrowing Cannot calculate PVR	Radiation dose, although this is less on modern scanners Fast heart rates can affect images, although can be mitigated with beta blockade Patient needs to be still, can now be achieved without anesthesia/sedation by use of wrap Limited definition of intracardiac structures Not routinely used for functional imaging Not dynamic Contrast is nephrotoxic	Expensive Artifact from stents, prosthetic valves and devices (MRI may be contraindicated in some) Contrast cannot be used in renal failure (but not required for majority of sequences) Usually requires at least sedation in infants and babies Lengthy acquisition and reporting
Uses	Baseline for standard full assessment at all stages in clinic, intraoperatively and post-operatively	Imaging pre-operatively to help define anatomy if concerns on echocardiography Review of lung parenchyma if concerns Assessment prior to SCPC Coronary artery imaging Where MRI is not possible due to pacemaker	Assessment prior to SCPC or TCPC Gold standard for RV function assessment Assessment of flow and quantification of valvar regurgitation, collateral flow, differential branch pulmonary artery flow

*AV, Atrioventricular; CT, computed tomography; LV, left ventricle; MRI, magnetic resonance imaging; PVR, pulmonary vascular resistance; RV, right ventricle; 3D, three dimensional; 2D, two dimensional; VA, ventriculoarterial*.

## Imaging Through the Stages

Table 2 summarizes imaging through the stages, and below each stage is addressed individually.

**Table 2:** Summary table of key imaging modalities at each stage.

**Prior to staged palliation**	**Prior to SCPC**	**Prior to TCPC**	**After TCPC**
Full segmental echocardiography Consider CT/MRI if concerns about the arch anatomy, branch pulmonary arteries or pulmonary or systemic venous drainage CT will also give information on lung fields if concerns over lymphangiectasia	Echocardiography PLUS CT/MRI/catheter to assess branch pulmonary arteries. Will also give information on adequacy of DKS and arch repair	Echocardiography PLUS (institutional preference) MRI with jugular venous pressure measurement to assess branch pulmonary artery anatomy and pulmonary pressures as well as quantify RV function and TR MRI with cardiac catheterisation derived pulmonary vascular resistance in higher risk patients (e.g., known respiratory pathology, previous atrial restriction, significant TR) Cardiac cathetersiation, particularly if intervention known to be needed	Echocardiography Consider CT/MRI if further information needed on Fontan circulation CT gives information on lungs additionally and is better for small collaterals and coronary arteries MRI allows quantification of flows and therefore calculations of collateral, fenestration, differential pulmonary artery flow as well as gold standard for function assessment

### Imaging Prior to Staged Palliation

The majority of infants with HLHS (>70%) are correctly antenatally diagnosed (6), and post-natal echocardiography confirms the diagnosis and the suitability for palliation. Some babies have an intact or highly restrictive atrial septum, which is considered a very significant risk factor as there may have been chronic pulmonary congestion *in utero* leading to lymphangiectasia (7). These babies can be born extremely sick and if an active management strategy is being planned, may need an immediate intervention (8). This may be an emergency surgical septectomy or NP but may also be an atrial septostomy.

Particular attention is paid to the competence of the tricuspid valve (TV) and RV function, as significant tricuspid regurgitation (TR) or RV dysfunction are risk factors for mortality (9–12). The atrial septum will be assessed in particular detail if the hybrid procedure (HP) is considered, as a decision will need to be made as to whether an atrial intervention is required before, during or after the procedure (atrial septal assessment is discussed in more detail below). The presence of aortic atresia with a diminutive aorta is considered by some groups to be a contraindication to HP, and a very tight isthmus may also become compromised (thus compromising coronary flow) with a ductal stent (13, 14). If there are any concerns over the great vessel anatomy, then a CT or MRI scan may provide further anatomical information to guide decision making (15).

### Imaging After Norwood Procedure

After NP, as well as ongoing assessment of the RV and valvar function, the operated areas must be interrogated for signs of function. Many patients may have epicardial (16) or transesophageal echocardiography (17, 18) in the operating theater, and if there are concerns either on the echocardiogram of with the patients' hemodynamics, then there should be a low threshold for diagnostic angiography ± intervention. For example in the patient with unexplained desaturation coming off bypass despite maximal oxygen therapy.

Prior to further surgical palliation, assessment is required to ensure adequate pulmonary artery size for SCPC. This article does not cover this area as this is pre-surgical assessment, but the assessment may take the form of cardiac CT, MRI or catheterization, with comparable outcomes with all modalities (19, 20).

#### Pulmonary Blood Flow

**Figure 2:**
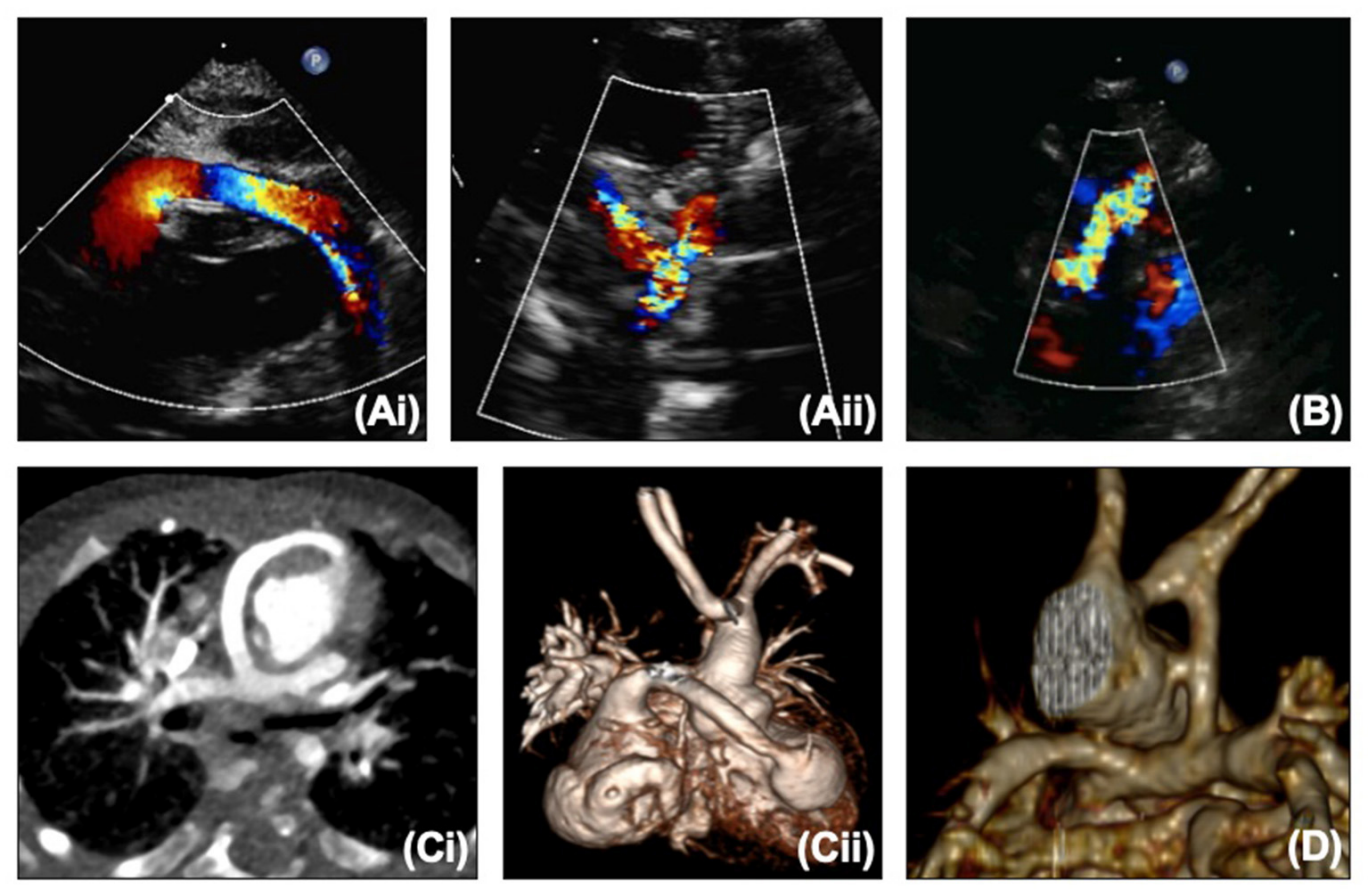
(**A**,i) Proximal and (**A**,ii) distal right ventricle to pulmonary artery conduit on echocardiography. **(B)** Modified Blalock-Taussig-Thomas shunt on echocardiography. (**C**,i,ii) Right ventricle to pulmonary artery conduit on CT. **(D)** Modified Blalock-Taussig-Thomas shunt on CT.

The BTTS and RV to PA conduit can both be seen on echocardiography (Figures 2A,B), but the RV to PA conduit can be quite anterior and therefore challenging to view along its length. The branch pulmonary arteries (PAs) can become distorted with torsion, tenting or stretching. Echocardiography may show narrowing on 2D or color of the shunt/conduit or branch PAs, or a point of flow acceleration may be noted. In a clinically unwell child with significant desaturation, this alone may be enough to move to cardiac catheterization, but in a more stable child, further information can be gained from CT or MRI (Figures 2C,D). CT can usually be performed without sedation in this age group, providing crisper images than MRI quicker, and without the use of anesthetic (which does not carry an insignificant risk in the HLHS group).

#### Damus-Kaye-Stansel

**Figure 3:**
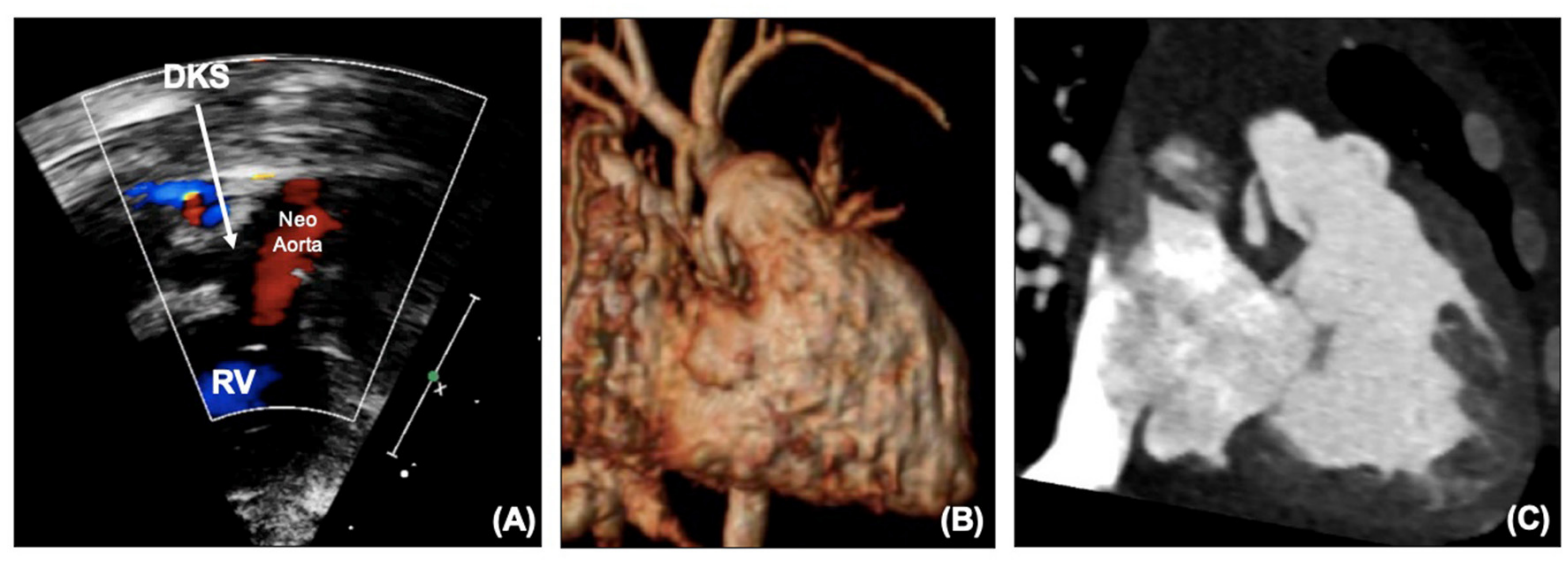
**(A)** DKS anastomosis on echocardiography. **(B)** DKS anastomosis on MRI. **(C)** DKS narrowing on CT. DKS, Damus-Kaye-Stansel; RV, right ventricle.

The coronary artery flow in HLHS patients is reliant on the DKS – blood leaves the heart through the neo-aortic valve into the aorta, with some then flowing retrogradely into the native aorta to the coronaries. Depending on the size of the native aorta, this connection can be a similar size to a coronary, effectively behaving like a single coronary origin. A patient who develops ischemic ECG changes, RV dysfunction or TR without arch obstruction needs to be considered as having obstruction to the DKS until it can be proven otherwise. Echocardiography can illustrate flow into the DKS (Figure 3A), and any turbulence needs to be considered obstruction. MRI can also visualize the DKS (Figure 3B) and CT imaging can acquire detailed views of the DKS and the coronary arteries to provide further imaging before considering whether an intervention is required (Figure 3C).

#### Reconstructed Arch

**Figure 4:**
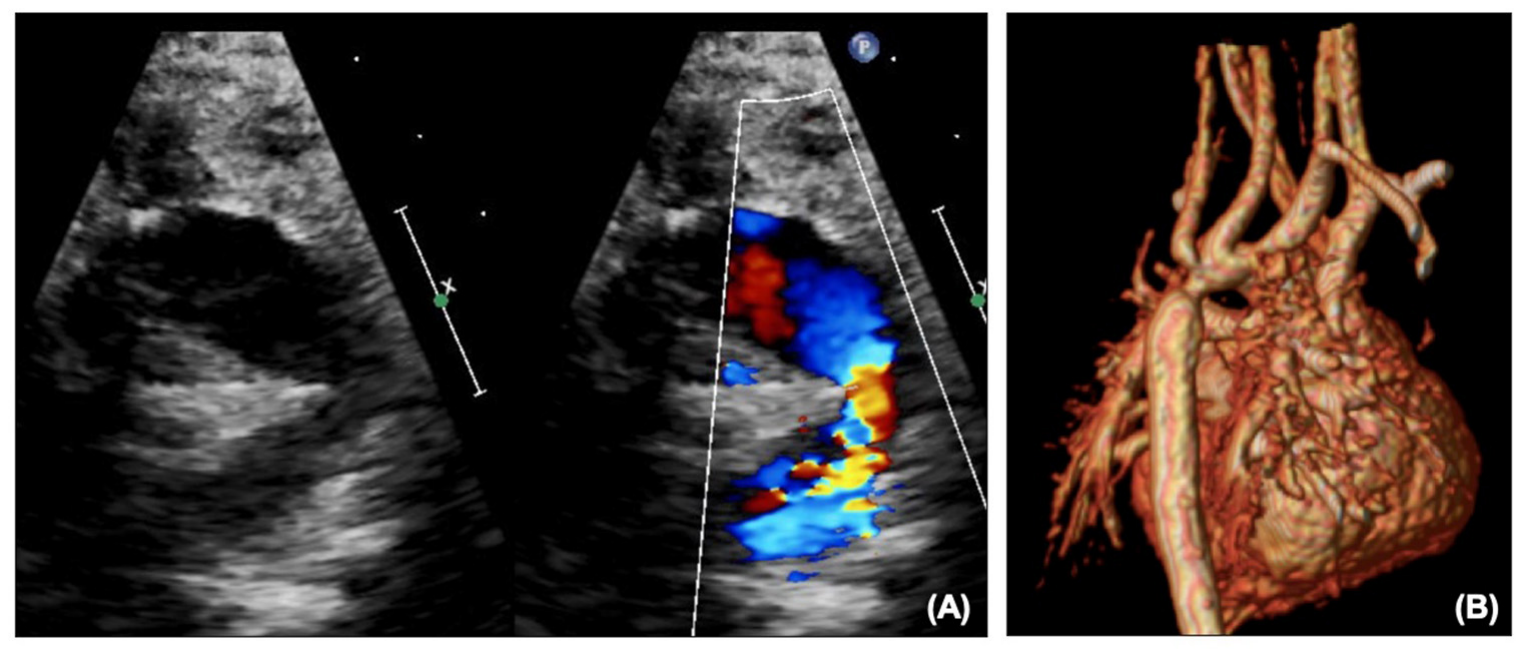
**(A)** Arch obstruction on echocardiography shown on 2D and color. **(B)** Arch obstruction on MRI.

The reconstructed aortic arch can be a source of obstruction, either with inadequate resection of ductal tissue leading to re-coarctation, or from torsion or twisting. This may be seen on echocardiography (Figure 4A) as turbulent flow with a Doppler pattern suggestive of coarctation, or presenting as impaired RV function and increasing TR. After NP, arch obstruction is critical, as not only does it increase the afterload to the systemic RV, but it may also increase pulmonary blood flow if it is easier to flow to the lungs than through the obstructed arch to the body, thus exacerbating the situation. Echocardiography alone may demonstrate the need for an intervention, but if the child is stable cross-sectional imaging with CT or MRI (Figure 4B) may further characterize the arch to decide whether the problem would be better tackled by a cardiac catheter intervention such as balloon or stent, or whether the arch needs reconstruction surgically.

#### Atrial Septum

In HLHS there is obstructed egress from the left atrium due to mitral stenosis or atresia. The atrial communication therefore needs to be widely patent unless deliberately left restrictive to try and “grow” the left heart. Hypoplastic left heart complex (HLHC) where there is potential for use of the left heart is a very different condition and requires extensive discussions separately and so is not discussed in this paper. At NP, there is usually an atrial septectomy, but the anatomy, particularly in those with a very small left atrium (LA) can be challenging and some atrial tissue or ridge may remain.

**Figure 5:**
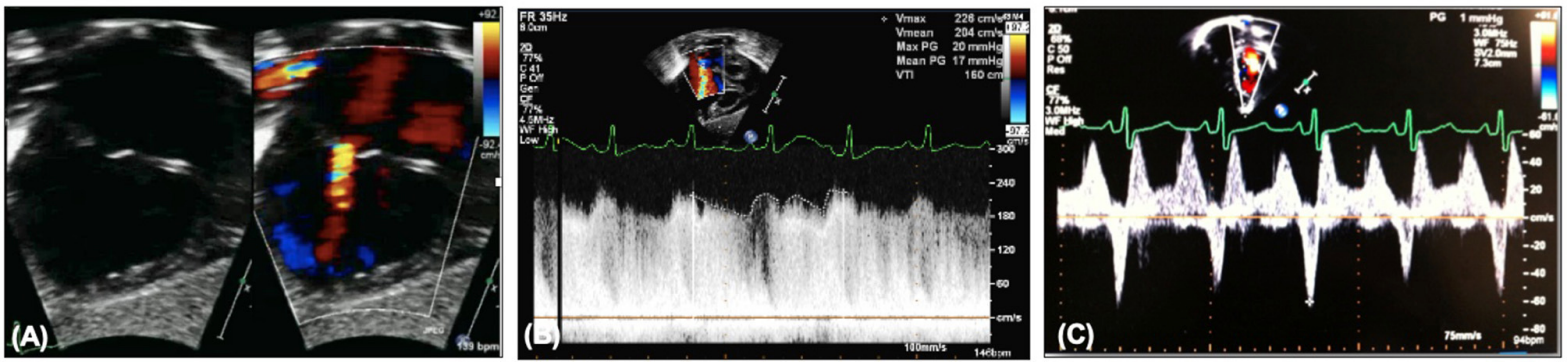
**(A)** Restrictive atrial communication on color. **(B)** Elevated transatrial Doppler mean gradient. **(C)** A wave reversal on pulmonary vein Doppler.

Echocardiography will demonstrate on 2D the atrial communication, and on color the flow should be low velocity and unrestricted (Figures 5A,B). Transatrial Doppler will estimate the pressure difference between the two atria, but will also be dependent on pulmonary flow, i.e., a high transatrial septal gradient may be related to high pulmonary blood flow and so arterial saturations should be part of the clinical picture. Another marker of increased left atrial pressure is A wave reversal on pulmonary vein Doppler (Figure 5C) as well as dilated pulmonary veins. 3D echocardiography may also be able to profile the atrial septum to assist in decision making for catheter atrial septostomy or atrial septal stenting.

### Imaging After Hybrid Procedure

After HP the key areas of interrogation in addition to RV and valvar function are the pulmonary artery bands and arterial duct (and ductal stent if *in situ*). Fenstermaker et al. describe the main areas of focus after HP which are also outlined below (21). Some patients may have undergone HP with borderline left hearts and will need comprehensive assessment to see if biventricular repair is possible (not covered within this article).

#### Pulmonary Artery Bands

**Figure 6:**
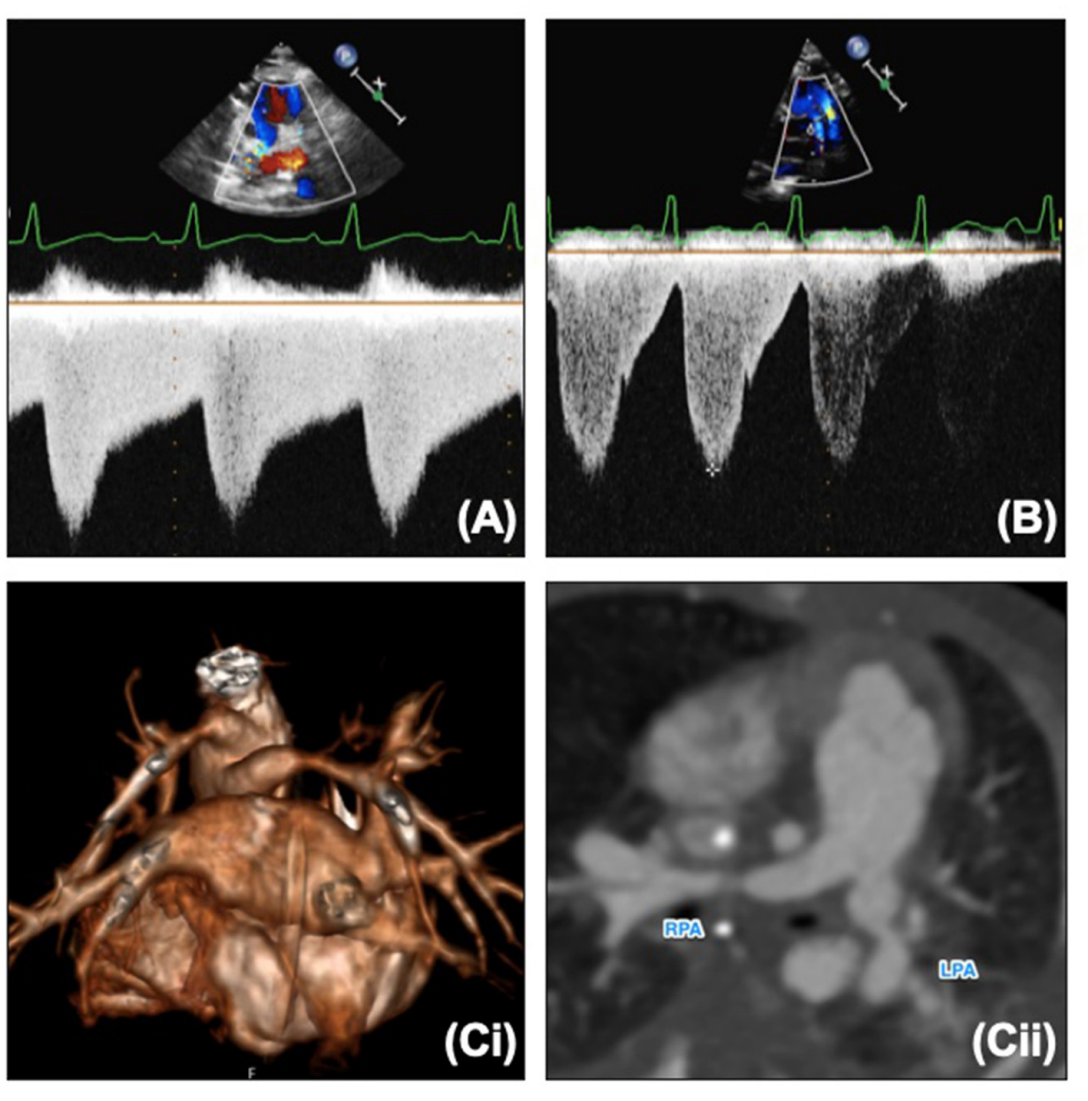
**(A)** Normal “sawtooth” pulmonary band Doppler pattern. **(B)** Abnormal “pulsatile” pulmonary band Doppler pattern. (**C**,i) 3D reconstruction of banded branch pulmonary arteries. (**C**,ii) Reformat of CT and banded branch pulmonary arteries. LPA, Left pulmonary artery; RPA, right pulmonary artery.

The pulmonary artery band position can be seen on echocardiography as a point of constriction with flow acceleration seen on color. The bands should not be too close to the bifurcation but should not be too far distally to avoid compromise of the hilar bifurcation. Each band should have Doppler interrogation and should produce a “sawtooth” pattern (Figure 6A). A “pulsatile” pattern (Figure 6B) with loss or reduction of diastolic flow may indicate a band that is too loose or that there is an increase in the distal pulmonary artery pressures (less diastolic flow as diastolic pressures are increased). The systolic velocity in isolation cannot be used to assess the pulmonary artery band. A low velocity may mean a loose band, or it could mean a high distal pulmonary artery pressure, and therefore should be used in conjunction with the clinical picture and chest x-ray. If the band is too loose there will likely be pulmonary plethora on the chest x-ray with a large heart. If there are high distal pressures the lung fields may look oligemic or certainly not plethoric. The velocities across the pulmonary artery bands will increase with time as expected as the child “grows out of” the bands. Pulmonary artery bands are usually addressed surgically, however in some older patients who are awaiting further growth prior to further surgery, balloon dilatation may be indicated, and further imaging with CT or MRI may give further information to aid planning (Figure 6C).

#### Atrial Septum

Assessment of the atrial septum is performed as described above. Additionally, it may be possible to gain further information from the pulmonary artery band Dopplers, presumed to be related to the effect of an increased left atrial pressure on the pulmonary artery pressures. The usual “sawtooth” pattern is lost, and the Doppler becomes more “pulsatile” with an increase in the pulsatility index and systolic to diastolic velocity ratio.

#### Guiding Atrial Septostomy

**Figure 7:**
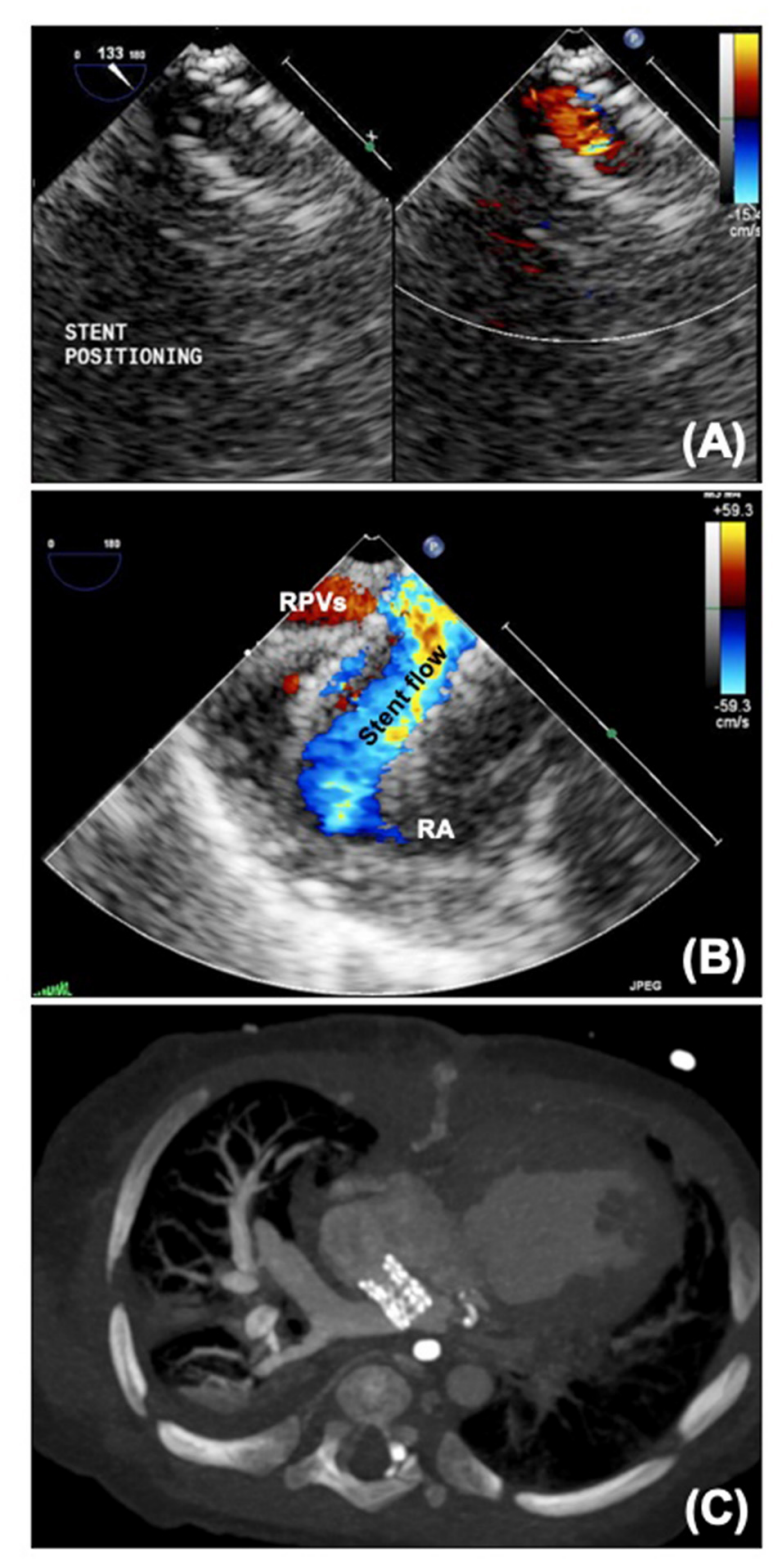
**(A)** Micro transoesophageal echocardiographic guidance of atrial septal stent. **(B)** Jailed pulmonary veins seen on echo. **(C)** CT scan showing atrial septal stent with dilated right pulmonary veins. RA, Right atrium; RPVs, right pulmonary veins.

Atrial septostomy in patients with HLHS can be extremely challenging due to the small size of the left atrium. It is therefore usually performed in the cardiac catheter laboratory under fluoroscopic guidance, but echocardiography can be a useful adjunct, particularly in guiding atrial septal stenting (Figure 7A). Transthoracic echocardiography can be used in a fashion similar to transposition of the great arteries, but transesophageal echocardiography, particularly now there is a very small “micro” probe available (18) may be easier for the interventional team as can be performed without interfering with the surgical field. Particular care needs to be taken with positioning, and in atrial septal stenting jailing of the right pulmonary veins may occur (Figures 7B,C), although flow through an open cell stent can still be good. In HLHS the aim is to make the atrial septum as unrestrictive as possible. As mentioned earlier, maintaining some preload to the left heart may be required in some patients with HLHC.

#### Interventional Takedown Hybrid

For some patients, HP bridges to potential biventricular circulation. In the absence of coarctation, and with adequate mitral and aortic valves, an interventional approach can be used to “take down” the hybrid. The arterial duct/ductal stent is occluded, and the pulmonary artery bands are ballooned, with further intervention performed on the aortic valve if necessary. Although this will be performed under fluoroscopy, echocardiographic assessment throughout can be complementary to the catheter data to review the adequacy of the circulation as well as aiding with dilatation of the aortic valve.

#### Arterial Duct

For patients maintained on a prostaglandin infusion, adequacy of the arterial duct should be assessed on echocardiography in the usual manner. Unless the management strategy is to assess the adequacy of the left heart as the duct closes (e.g., in borderline left hearts), it should remain widely patent with no significant constriction seen on 2D, color or Doppler. For patients with aortic atresia, coronary blood flow is also duct dependent.

**Figure 8:**
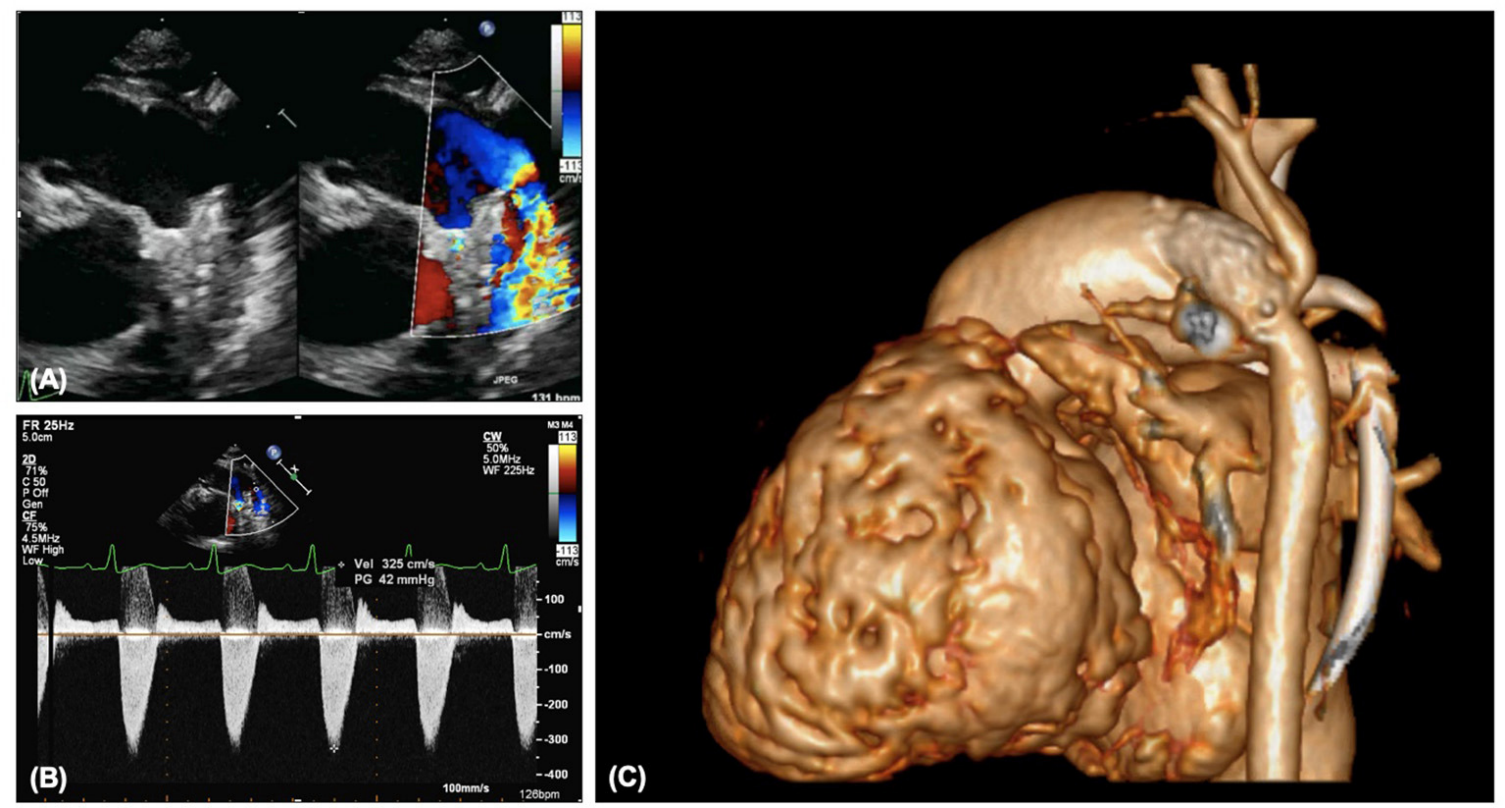
**(A)** Narrowing of arterial ductal stent on 2D and color. **(B)** Increased Doppler gradient on ductal stent. **(C)** Constriction distal to the ductal stent with involvement of the isthmus and retrograde aortic arch.

In patients with a ductal stent any narrowing should be noted on 2D and color (Figure 8A). Doppler interrogation (Figure 8B) should occur proximal to the stent in the main pulmonary artery, in the proximal portion of the stent, in the mid stent, in the distal stent and distal to the stent in the descending aorta. Any step-up in velocity should be documented, and if further information is required cardiac CT can provide additional information but does not always show the extent of neo-intimal proliferation (Figure 8C). Over time the ductal stent velocities will increase, but as with residual arch obstruction, even a mild degree of narrowing can be clinically significant in HLHS. The degree of flow reversal on the Doppler reflects the “steal” and if there is significant flow reversal associated with signs of systemic or coronary underperfusion (e.g., reduced function, ECG changes) then there may need to be consideration to further reduce the systemic vascular resistance or tighten the pulmonary artery bands.

The isthmal area and retrograde flow into the aortic arch are often challenging to see on echocardiography, but any increase in velocity into this area, particularly in patients with aortic atresia is likely to be significant and warrant further imaging or catheter intervention.

### Imaging After Superior Cavopulmonary Anastomosis

**Figure 9:**
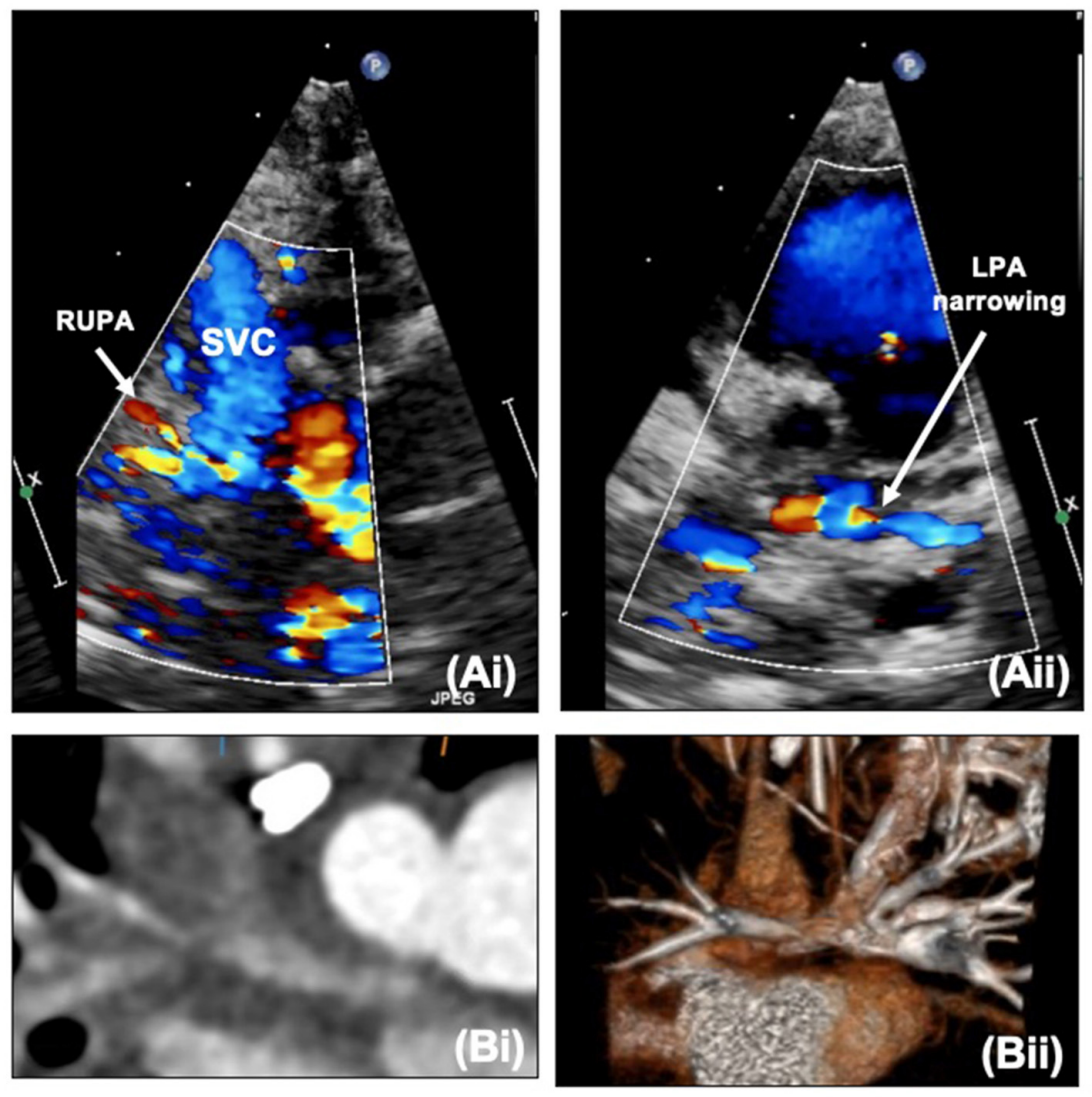
(**A**,i) Right upper pulmonary artery narrowing with Glenn on echocardiography. (**A**,ii) Proximal left pulmonary artery narrowing – site of previous pulmonary artery band. (**B**,i) Narrowing of the right pulmonary artery at the bifurcation. (**B**,ii) Proximal left pulmonary artery narrowing on CT. LPA, Left pulmonary artery; RUPA, right upper pulmonary artery; SVC, superior caval vein.

Along with ongoing assessment of the RV and valvar function as well as the atrial communication, DKS and arch, the main foci of assessment after SCPC are the branch pulmonary arteries and caval anastomosis. With increasing age (and decreasing compliance) echocardiographic views become more challenging and cross-sectional imaging with cardiac CT or MRI may be useful to define the pulmonary arterial anatomy further. Figure 9 outlines images of the branch pulmonary arteries by echocardiography and CT. Cardiac CT gives crisp images as well as showing well the relationship between the branch pulmonary arteries and the airways, in particular the left main bronchus which could potentially become compromised with left pulmonary artery stenting. Cardiac MRI allows calculation of differential flow through each branch pulmonary artery to aid in decision making as well as estimating collateral flow. Both modalities will allow demonstration of venovenous and aortopulmonary collaterals – these can be missed on both modalities if there is a right arm injection with an early acquisition.

Assessment prior to TCPC is not described in detail here but can be performed using cardiac catheterization or MRI with measurement of jugular vein pressure as surrogate of pulmonary pressures with equivalent outcomes (22). If there are concerns on MRI with pressure measurement, then the patient may require full assessment of the pulmonary vascular resistance via cardiac catheterization or cardiac catheterization with MRI. MRI cardiac catheterization gives the advantage of using measured rather than derived flow, thus increasing the reliability of the calculation.

### Imaging After Total Cavopulmonary Connection

**Figure 10:**
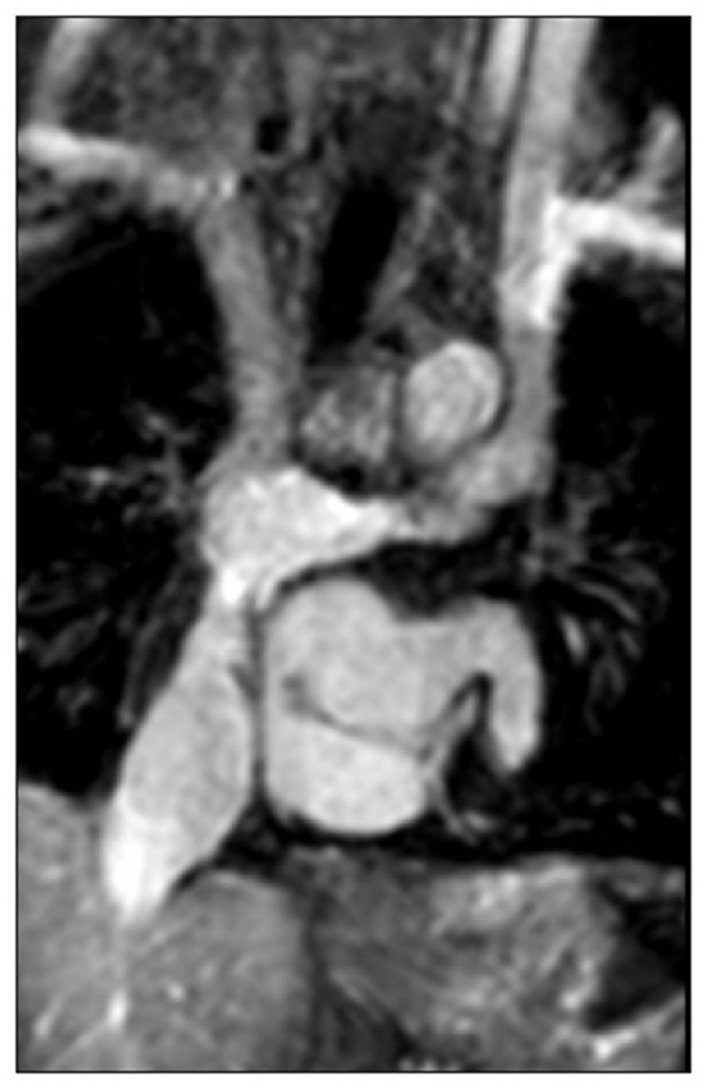
Narrowing of the Fontan circuit seen on MRI.

After TCPC there should be continued review of the operated areas and RV and atrioventricular valvar function. The branch pulmonary arteries remain an area of concern and can continue to be assessed as above, usually more with CT/MRI as the acoustic windows worsen with age even if compliance increases. The inferior caval connection and tunnel now also need to be assessed, with phasic flow expected. If there is reduced distensibility of the IVC with dilatation and spontaneous contrast, suspicions should be raised of increased pressures. These pressures could be related to elevated pulmonary vascular resistance or high left atrial pressure, but they could also point to a narrowing in the TCPC circuit or thrombus (Figure 10).

#### Fenestration

Creation of a fenestration in the TCPC varies by institution but is particularly necessary when there are concerns over the transpulmonary gradient or branch pulmonary artery narrowing. The communication between the Fontan circuit and atrial mass acts as a “blow off” valve if the Fontan pressures are increased, at the expense of saturations. Patency of the fenestration can be seen as flow on echocardiography, and Doppler interrogation will estimate the transpulmonary gradient and can be useful to monitor patients.

**Figure 11:**
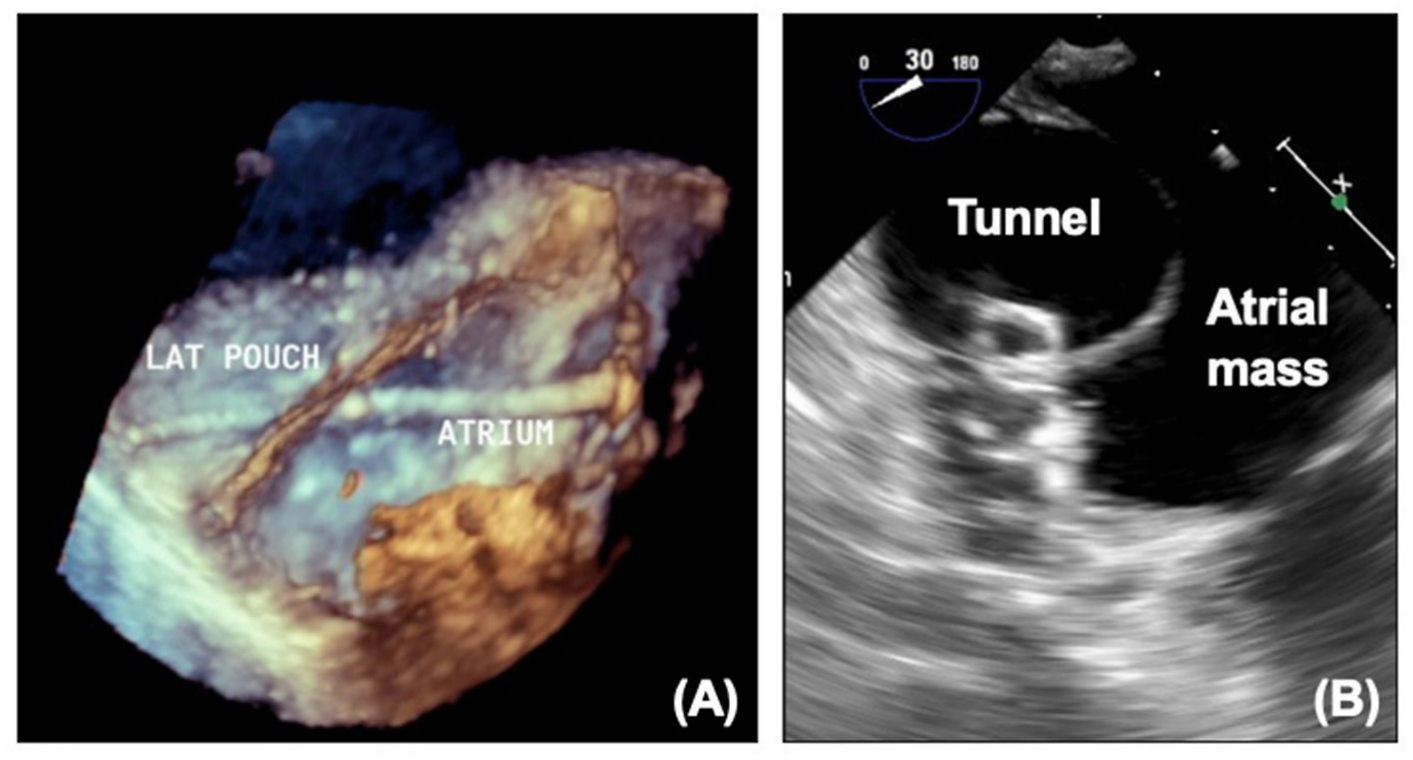
**(A)** 3D echocardiogram showing wire across fenestration. **(B)** Device in fenestration seen on echocardiography. LAT, Lateral tunnel pouch.

For some patients who have satisfactory hemodynamics, the fenestration may be a source of unneeded desaturation or thrombus and interventional closure of the fenestration with a device may be indicated. Proportion of fenestration flow can be calculated on MRI, and this can be used in association with invasive pressure measurements to decide whether closure is appropriate. There will often be test occlusion in the catheter laboratory prior to complete occlusion with re-assessment of the pressures. Transesophageal echocardiography can aid the interventional team with crossing the fenestration and guiding device closure (Figure 11).

#### Pulmonary Veins

**Figure 12:**
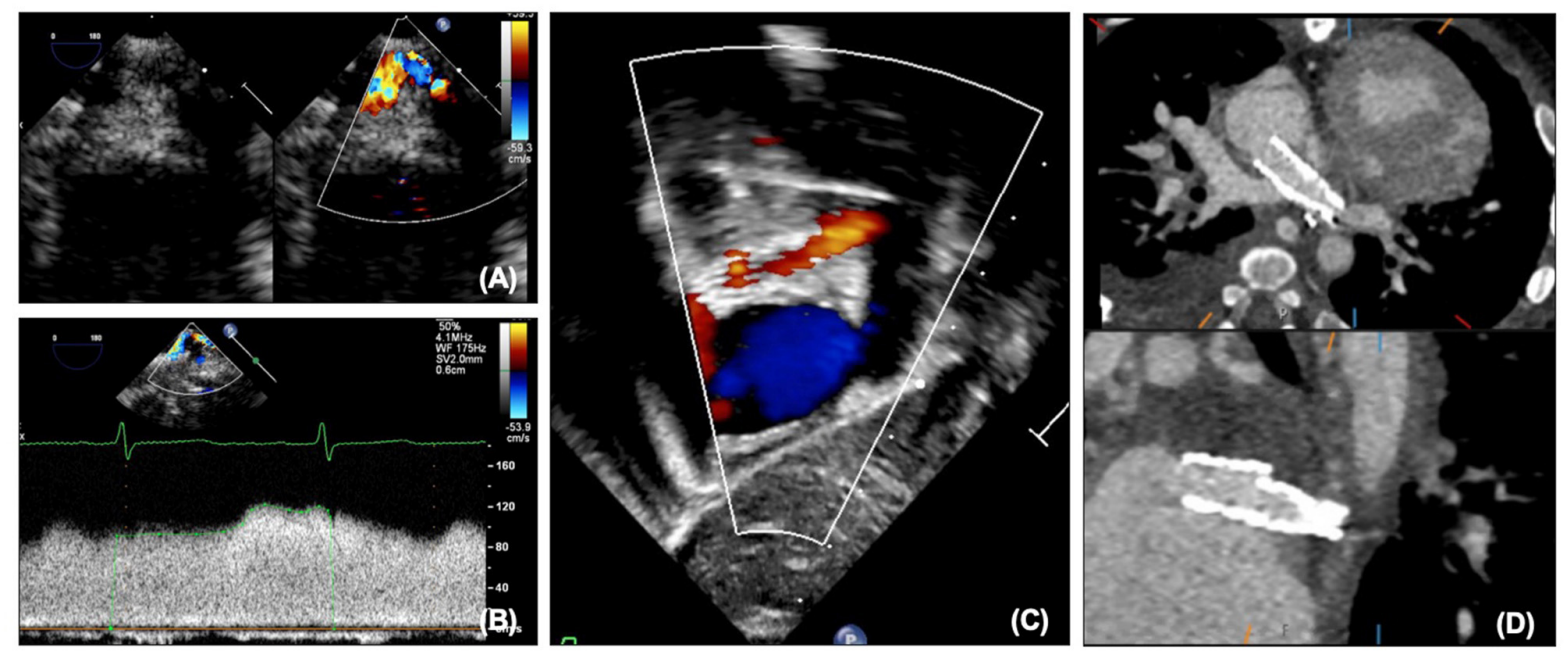
**(A)** Pulmonary vein stenosis on echocardiography on 2D and color. **(B)** Abnormal pulmonary vein Doppler due to stenosis. **(C)** Left pulmonary vein and atrial septal stents on transthoracic echocardiography. **(D)** Left pulmonary vein and atrial septal stents on CT.

Although for most patients with HLHS the left pulmonary veins drain normally to the left atrium, abnormalities of pulmonary venous drainage can be seen, and over time unfortunately pulmonary vein stenosis and sclerosis can be seen. This can be assessed with echocardiography on 2D, color and Doppler (Figures 12A,B). Although like the atrial communication, at earlier stages pulmonary venous flow can be affected by pulmonary blood flow, after SCPC pulmonary blood flow is not significantly increased and therefore increased velocities are more likely to truly represent stenosis. Caution must also be taken in patients with significant branch pulmonary stenosis, as the return in the ipsilateral pulmonary vein may also be reduced thus underestimating stenosis. As surgical intervention on pulmonary veins has significant limitations, stenting of the pulmonary veins may be considered, and can be performed under fluoroscopy with additional information from transesophageal echocardiography and assessed afterwards on transthoracic and CT (Figures 12C,D).

#### Lymphatics

**Figure 13:**
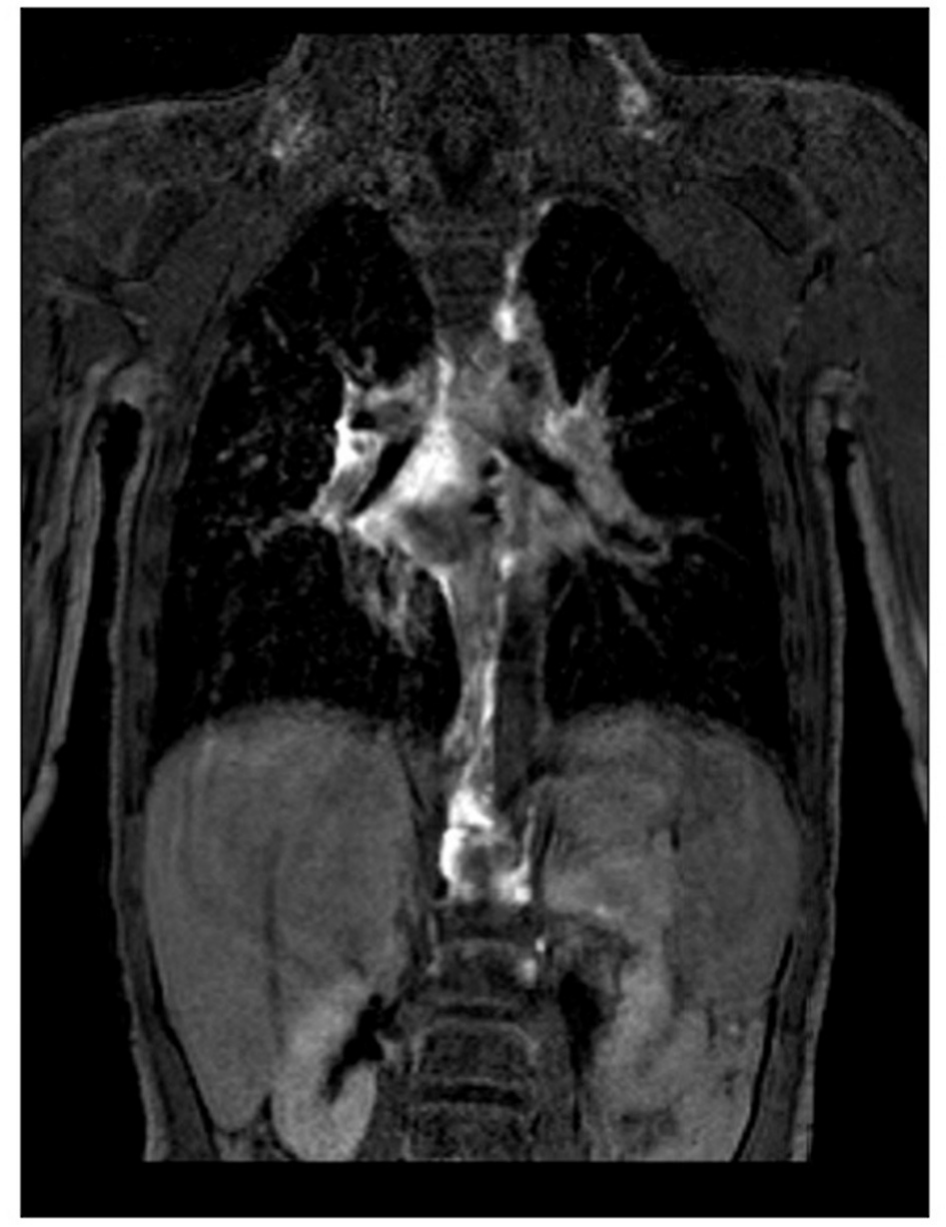
MRI lymphatic imaging.

Plastic bronchitis and protein losing enteropathy are life-threatening complications that can occur during single ventricle palliation. The role of the lymphatics in these conditions is currently under intense review. Cardiac MRI provides a noninvasive modality that can delineate the lymphatic drainage and potential foci for intervention (Figure 13) (23–25).

#### Thrombus

**Figure 14:**
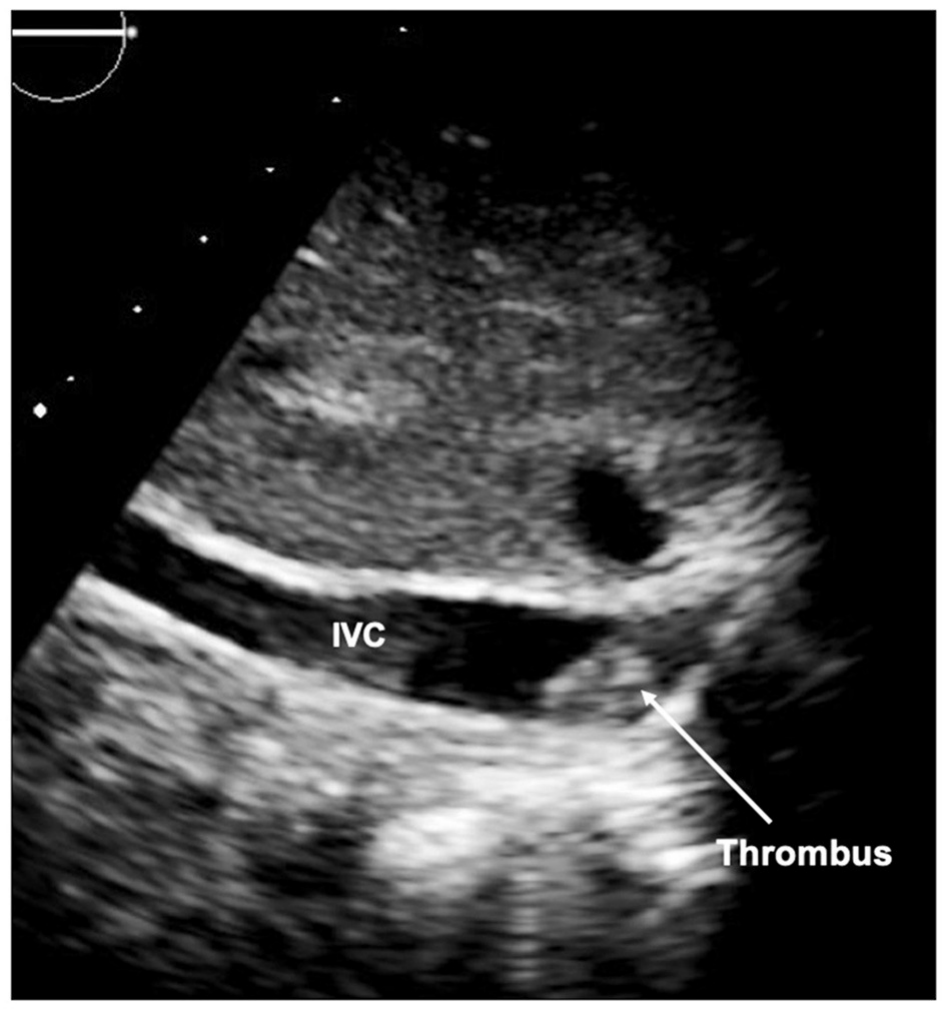
Thrombus seen in inferior limb of the Fontan circuit at the inferior caval vein (IVC) junction.

A further peril of the single ventricle pathway is thrombus (Figure 14). As the circulation relies on unobstructed flow through the circuit to the pulmonary vascular bed, any thrombus can be life threatening. Thrombus can be identified on echocardiography (transesophageal echocardiography may be required), but may also be detected on CT or MRI, particularly in patients with poor acoustic windows. Catheter intervention with thrombolysis or thrombectomy may be required and the pre-procedural imaging will give an outline of the potential target areas.

## Conclusions

Multimodality imaging is essential prior to and throughout staged palliation for HLHS. The different modalities each carry their own advantages and disadvantages and complement each other well. Not only can imaging be used to assess before and after procedures, but also to help guide interventions in this very complex group of patients.

## Author Contributions

The author confirms being the sole contributor of this work and has approved it for publication.

## Conflict of Interest

The author declares that the research was conducted in the absence of any commercial or financial relationships that could be construed as a potential conflict of interest.
